# Folic acid supplements and colorectal cancer risk: meta-analysis of randomized controlled trials

**DOI:** 10.1038/srep12044

**Published:** 2015-07-01

**Authors:** Tingting Qin, Mulong Du, Haina Du, Yongqian Shu, Meilin Wang, Lingjun Zhu

**Affiliations:** 1 Department of Oncology, The First Affiliated Hospital of Nanjing Medical University, Nanjing, China; 2 Department of Environmental Genomics, Jiangsu Key Laboratory of Cancer Biomarkers, Prevention and Treatment, Cancer Center, Nanjing Medical University, Nanjing, China; 3 Department of Genetic Toxicology, the Key Laboratory of Modern Toxicology of Ministry of Education, School of Public Health, Nanjing Medical University, Nanjing, China

## Abstract

Numerous studies have investigated the effects of folic acid supplementation on colorectal cancer risk, but conflicting results were reported. We herein performed a meta-analysis based on relevant studies to reach a more definitive conclusion. The PubMed and Embase databases were searched for quality randomized controlled trials (RCTs) published before October 2014. Eight articles met the inclusion criteria and were subsequently analyzed. The results suggested that folic acid treatment was not associated with colorectal cancer risk in the total population (relative risk [RR] = 1.00, 95% confidence interval [CI] = 0.82–1.22, *P* = 0.974). Moreover, no statistical effect was identified in further subgroup analyses stratified by ethnicity, gender, body mass index (BMI) and potential confounding factors. No significant heterogeneity or publication bias was observed. In conclusion, our meta-analysis demonstrated that folic acid supplementation had no effect on colorectal cancer risk. However, this finding must be validated by further large studies.

Folic acid is a water-soluble vitamin first extracted and purified in 1941 from spinach leaves. Folic acid deficiency causes an imbalance in the one-carbon metabolic pathway, which is vital to hemoglobin synthesis as well as DNA synthesis, repair and methylation^1^,^2^. Research on folic acid treatment traces back to the last century when Metz *et al.* reported that pregnant women received iron from folic acid supplementation^3^. Dr. Laurence was a forerunner exploring the association between folic acid and neural tube defects^4^. Later, folic acid was reported to influence public health conditions, such as cardiovascular diseases, acute lymphoblastic leukemia, neuropathy and cancers, including colorectal cancer^5^,^6^,^7^,^8^,^9^.

Colorectal cancer is one of the most aggressive cancers worldwide, with mortality increasing in recent years^10^,^11^. Despite new therapeutic approaches, the prognosis of patients with colorectal cancer remains poor, and the median survival is only approximately 20 months for individuals with advanced disease^12^. Therefore, the need to discover proper chemopreventive agents to relieve disease burden is urgent. One potential target for therapy involves aberrant methylation, which is associated with the pathogenesis of early-stage colorectal cancer. Given that folic acid affects DNA methylation, it may play a role in carcinogenesis^13^.

Many researchers have examined the potential effects of folic acid supplementation in the prevention of colorectal cancer^14^. Various studies have focused on the association between folic acid and colorectal cancer for approximately two decades^15^, but existing epidemiological data are inconsistent. Folic acid fortification may increase the rate of colorectal cancer^16^. However, a meta-analysis of three randomized controlled trials (RCTs) observed no such effect^17^. Given that the results of the latest RCTs have been inconsistent, we performed this meta-analysis to provide a systematic evaluation.

## Results

### Study characteristics

**Figure 1:**
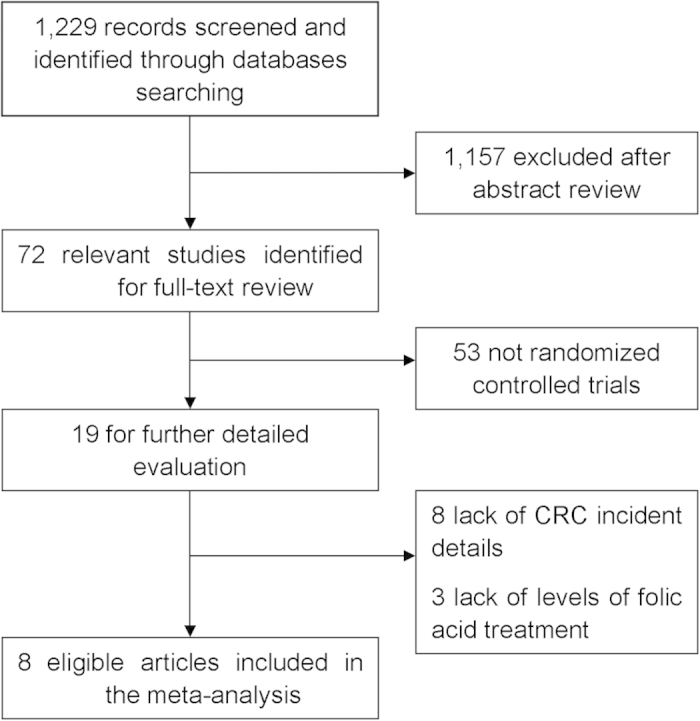
A flow chart of the study identification and selection.

A total of 1,229 relevant reports were retrieved from the PubMed and Embase databases, and 72 eligible studies were identified for further assessment. Eight RCTs ultimately met the inclusion criteria^18^,^19^,^20^,^21^,^22^,^23^,^24^,^25^ (Fig. 1), two of which were related to the prevention of cardiovascular disease^18^,^23^, four were related to the occurrence or recurrence of colorectal adenoma^19^,^20^,^22^,^23^ and two studies assessed cancer risk^21^,^24^. Each study was a population-based RCT, which ensured the methodological quality of the article. All trials were placebo-controlled except for the studies by Gao *et al.* and Logan *et al.*^20^,^25^. Seven studies were double-blinded studies, whereas the remaining one provided no details regarding this^25^. The dose of folic acid supplemented daily varied from 0.5 to 2.5 mg. Each article was of high quality based on our quality assessment, and all had received a score ranging from 3 to 5 out of a total of 5 points. Detailed characteristics of the relevant literature are presented in Table 1.

### Quantitative synthesis

**Figure 2:**
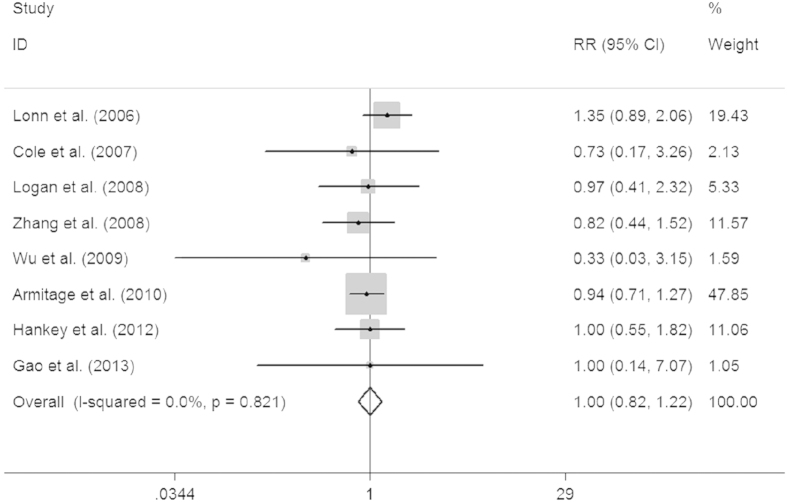
Forest plot of the association between colorectal cancer risk and folic acid supplementation. The squares and horizontal lines correspond to the study-specific RR and 95% CI, respectively. The areas of the squares reflect the weight. The diamond represents the summary RR and 95% CI.

Our analysis revealed that supplementary folic acid lacked any association with the colorectal cancer incidence (relative risk [RR] = 1.00, 95% confidence interval [CI] = 0.82–1.22, *P* = 0.974; Fig. 2). A subgroup analysis based on ethnicity led to a similar conclusion (Caucasian RR = 0.91, 95% CI = 0.71–1.17, *P* = 0.463; mixed ethnicity RR = 1.19, 95% CI = 0.85–1.67, *P* = 0.303). In further analyses stratified by age, gender, body mass index (BMI), dose of folic acid, duration of the study or putative confounding factors, no significant effect was observed (Table 2).

### Tests for heterogeneity and sensitivity

Fixed-effects models were utilized to analyze the association because no significant heterogeneity was observed (Table 2). The sensitivity analyses revealed that the RR with 95% CI was not obviously affected by removing one article at a time (data not shown).

### Publication bias

**Figure 3:**
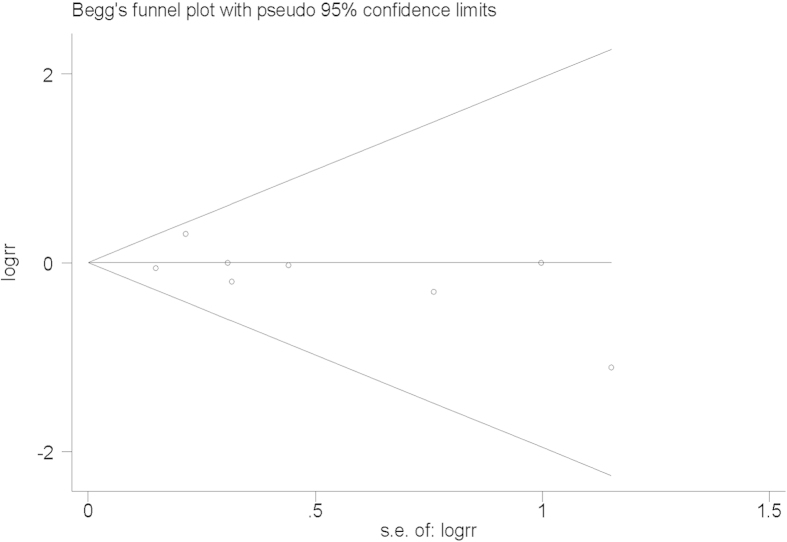
Begg’s funnel plot for the publication bias test. Each point represents a separate study for the indicated association.

The shape of Begg’s funnel plot did not exhibit any obvious asymmetry (Fig. 3), and the Egger’s test revealed no evidence of publication bias (*t* = −1.05, *P* = 0.334).

## Discussion

Folic acid was confirmed to protect against neural tube defects (NTDs) in the early 1990s. Since then, folic acid has been recommended to women of childbearing age to prevent birth defects^26^,^27^. Considerable attention has focused on the potential role of folic acid in preventing carcinogenesis, owing to its functions in DNA synthesis, repair and methylation^28^. For example, an association study by Lashner *et al.* explored the impact of folic acid treatment on cancer incidence in patients with chronic ulcerative colitis^29^. A meta-analysis by Sanjoaquin *et al.* suggested a small protective function of folic acid consumption on colorectal cancer^30^. Another meta-analysis by Kennedy *et al.* revealed a reduced cancer risk for subjects with increased folic acid intake^31^. In our analyses, however, no specific evidence for an overall relationship was detected. The inclusion criteria were potentially responsible for the difference, as only RCTs were included in our meta-analysis.

A high folic acid level was reported to break the homeostasis of the one-carbon metabolic pathway and increase cancer risk^32^, and fluorouracil misincorporation and DNA methylation disorders were postulated as possible mechanisms^33^. However, a subgroup analysis based on folic acid level did not change our conclusion in this study. The occurrence and development of colorectal cancer are associated with complex processes that may persist for 20 years or longer^34^, and longer duration trials would likely be needed to detect clinically detectable effects. In our analyses, however, a longer duration of treatment (>mean) did not exhibit a difference compared with a shorter (<mean) treatment period. Because the adenoma-carcinoma sequence is widely accepted as a gradual progression consisting of original normal epithelial mucosa, adenoma and ultimately carcinoma^35^, the existence of colorectal adenoma before the randomized trial could have been at least partly responsible for the formation of colorectal cancer in the above studies. In the present study, three articles were analyzed after stratification based on the prior existence of adenoma, but no statistically significant difference was observed based on a prior adenoma in these cases; however, the number of patients was relatively small^19^,^20^,^22^. We also observed no differences in the subgroup analysis based on gender. We hypothesized that the duration of follow-up influenced the apparent effect, but the corresponding analysis could not be performed given the lack of available information.

Previous animal experiments suggested that the effect of folic acid on carcinogenesis is dependent on the supplementary dose. In previous studies of normal cells, folic acid deficiency enhanced carcinogenesis, whereas supplementation enhanced tumor progression in tumor cells^36^,^37^. Based on our present results, both higher (>mean) and lower (<mean) doses did not affect the risk. Interestingly, one trial included in our meta-analysis suggested that folic acid plasma levels were more important than supplement levels^25^. In addition, a recent study demonstrated that the plasma folic acid concentration was associated with the risk of colorectal cancer, particularly for individuals with precancerous lesions^38^. However, details regarding dietary and plasma folic acid levels were not available for the studies included in our analyses, limiting our ability to assess the correlation. Elimination of subjects with risk factors (smoking, hypertension, alcohol intake, diabetes, etc.) would cause the data to be insufficient. Thus, these records were included, and subgroup analyses were conducted. However, all of these analyses yielded negative results (Table 2).

Despite the diversity of studies concerning different populations, family history, living environments, habits and customs, no significant heterogeneity or publication bias was observed in our analysis. In general, the articles included were compatible for this meta-analysis. To our knowledge, this meta-analysis is the most systematic examination of the association between folic acid supplementation and colorectal cancer based on all relevant RCTs. All chosen articles were of high quality, thus enhancing the reliability of our analyses and reducing the inherent bias. Such analyses may offer hypotheses for further functional studies and may shed light on the complexities of the pathways involved in colorectal cancer development. There are also some limitations that should be kept in mind when interpreting the results. First, two articles had relatively small samples, which may have affected the conclusion^22^,^25^. Second, potential heterogeneity may have been introduced due to methodological differences among trials. Finally, the possibility of publication bias existed in the review process, which could cause misleading results.

In conclusion, the present meta-analysis, which included the largest number of relevant RCTs to date, indicated that folic acid supplementation did not affect the colorectal cancer risk. However, questions remain regarding the role folic acid may play in colorectal cancer prevention, and larger studies with a rigorous design and strict methods are needed.

## Methods

### Publication search

We searched the PubMed and Embase databases for all studies published before October 2014. Combinations of the following MeSH terms were used for the search: “folic acid/folate,” “colorectal cancer,” “colon/rectal cancer” and “carcinoma.” Articles including association studies between folic acid fortification and colorectal cancer incidence were collected. The reference lists of relevant studies were also reviewed to identify any studies that were potentially missed. To be eligible for our analysis, the studies had to meet the following criteria: 1) exclusive RCT design; 2) explores the correlation between folic acid supplementation and colorectal cancer risk; 3) RR with a 95% CI or the number of colorectal cancer events was reported; 4) the supplementary folic acid level was stated; 5) published in English. Figure 1 provides a flow chart of the selection procedure.

### Data extraction and quality assessment

Two authors screened the relevant publications and then extracted all data independently, complying with the selection criteria above. Discrepancies were resolved by another author after group discussion. The following data were extracted: first author’s last name, year of publication, ethnicity of the subjects, source of controls, sample size, age, gender, BMI, smoking status, prior disease, folic acid intake level, additional treatment, duration of the studies and colorectal cancer incidence. Ethnicity was categorized as Asian, African, Caucasian or mixed.

Quality assessments were performed based on the following features: randomization, double-blinding, generation of random numbers, reporting of dropouts and withdrawals and concealment of allocation^39^. Each feature was awarded one point, and all studies scored between 0 and 5 (see Supplementary Table S1 online). The publications that received a score greater than 2 were considered to be of high quality.

### Statistical analysis

The relative risk (RR) with a 95% confidence interval (CI) was calculated to measure the strength of the association. The presence of between-trial heterogeneity was tested by the *χ*^*2*^-based Q test. The degree of variability was assessed by calculating the *I*^*2*^(inconsistency index) value. If the result of the Q test was *P* > 0.10, the RR was analyzed by the fixed-effects model. Otherwise, a random-effects model was used due to significant heterogeneity. A sensitivity analysis was performed to estimate the stability of the results by removing each study from the analysis, one at a time. Potential publication biases were also evaluated. In addition to visual inspection of the funnel plot, a value of *P* < 0.05 was considered to indicate the presence of significant publication bias^40^. All analyses were performed using the Stata software program (version 10.1; Stata Corporation, College Station, Texas) using two-sided *P-*values.

## Additional Information

**How to cite this article**: Qin, T. *et al.* Folic acid supplements and colorectal cancer risk: meta-analysis of randomized controlled trials. *Sci. Rep.* **5**, 12044; doi: 10.1038/srep12044 (2015).

## Supplementary Material

Supplementary Table S1

## Figures and Tables

**Table 1:** Main characteristics of studies pooled in this meta-analysis.

				Sample size										CRC incidents		
Author	Year	Ethnicity	Control source	Active	control	Age (year)	Male (%)	BMI (kg/m^2^)	Current smoker (%)	Prior disease (daily)	Folic acid	Additional treatment	Duration (months)	Active	control	Score of quality
Lonn *et al.*^19^	2006	Mixed	HB	2758	2764	68.9	71.8	29.7	11.5	Vascular disease or diabetes	2.5 mg	50 mg vitamin B_6_ and 1 mg vitamin B_12_	60	50	37	5
Cole *et al.*^20^	2007	Mixed	HB	516	505	57.0	63.8	27.5	14.5	Colorectal adenoma	1 mg	81 mg or 325 mg aspirin or none	75	3	4	5
Logan *et al.*^21^	2008	Caucasian	HB	432	421	NR	NR	NR	NR	Colorectal adenoma	0.5 mg	300 mg aspirin or none	27	10	10	5
Zhang *et al.*^22^	2008	Caucasian	HB	2721	2721	62.8	0	30.6	11.9	CVD or 3 or more coronary risk factors	2.5 mg	50 mg vitamin B_6_ and 1 mg vitamin B_12_	88	18	22	3
Wu *et al.*^23^	2009	Caucasian	HB	338	334	65.3	38.4	25.7	7.0	Colorectal adenoma	1 mg	None	57	1	3	5
Armitage *et al.*^24^	2010	Caucasian	HB	6033	6031	64.2	83.0	NR	12.0	MI, other CHD, other vascular disease or diabetes	2 mg	1 mg vitamin B_12_	80	86	91	5
Hankey *et al.*^25^	2012	Mixed	HB	4089	4075	NR	63.9	NR	23.3	Stroke or transient ischemic attack	2.5 mg	50 mg vitamin B_6_ and 1 mg vitamin B_12_	41	21	21	5
Gao *et al.*^26^	2013	Asian	HB	430	430	60.5	50.3	NR	17.3	None	1 mg	None	36	2	2	4

HB, hospital based; BMI, body mass index; CVD, cardiovascular disease; MI, myocardial infarction; CHD, coronary heart disease; CRC, colorectal cancer; NR, not reported.

**Table 2:** Summary of overall and subgroup analyses of the association between folic acid treatment and colorectal cancer risk.

	CRC incidents						
Stratification variables^a^	Active	Control	*RR (95% CI)*	*Z*	*P>*	*P_h_*	*I^2^*(%)
Total population	191	190	1.00 (0.82–1.22)	0.03	0.974	0.821	<0.001
Ethnicity							
Caucasian	115	126	0.91 (0.71–1.17)	0.73	0.463	0.807	<0.001
Mixed	74	62	1.19 (0.85–1.67)	1.03	0.303	0.578	<0.001
Total	189	188	1.00 (0.82–1.23)	0.03	0.974	0.727	<0.001
Age (year)^b^							
<mean^20^,^22^,^24^,^26^	109	119	0.92 (0.71–1.19)	0.67	0.501	0.967	<0.001
>mean^19^,^23^	51	40	1.28 (0.85–1.93)	1.17	0.243	0.227	31.4
Total	160	159	1.01 (0.81–1.25)	0.05	0.957	0.605	<0.001
Male (%)^b^							
<mean^22^,^23^,^25^	22	27	0.78 (0.44–1.37)	0.87	0.384	0.724	<0.001
>mean^19^,^20^,^24^,^25^	160	153	1.05 (0.84–1.30)	0.39	0.694	0.542	<0.001
Total	182	180	1.01 (0.82–1.23)	0.05	0.962	0.727	<0.001
BMI (kg/m2 b							
<mean^20^,^23^	4	7	0.56 (0.17–1.91)	0.92	0.355	0.561	<0.001
>mean^19^,^22^	68	59	1.15 (0.82–1.63)	0.81	0.417	0.188	42.3
Total	72	66	1.09 (0.78–1.52)	0.51	0.608	0.364	5.9
Dose of folic acid^b^							
<mean^20^,^21^,^23^,^24^,^26^	102	110	0.92 (0.71–1.21)	0.58	0.562	0.919	<0.001
>mean^19^,^22^,^25^	89	80	1.11 (0.82–1.50)	0.70	0.485	0.386	<0.001
Total	191	190	1.00 (0.82–1.22)	0.03	0.974	0.821	<0.001
Duration^b^							
<mean^19^,^21^,^23^,^25^,^26^	84	73	1.15 (0.84–1.57)	0.86	0.389	0.711	<0.001
>mean^20^,^22^,^24^	107	117	0.91 (0.70–1.19)	0.68	0.497	0.881	<0.001
Total	191	190	1.00 (0.82–1.22)	0.03	0.974	0.821	<0.001
Prior Disease							
Colorectal adenoma^19^,^20^,^23^	14	17	0.81 (0.40–1.62)	0.61	0.544	0.669	<0.001
CVD^19^,^22^,^24^,^25^	175	171	1.02 (0.83–1.26)	0.22	0.829	0.477	<0.001
Possible confounding factors							
Vitamin^19^,^20^,^22^,^23^,^24^,^25^	179	178	1.01 (0.82–1.24)	0.05	0.962	0.605	<0.001
Antiplatelet drugs^19^,^20^,^21^,^23^,^25^	85	75	1.13 (0.83–1.54)	0.77	0.442	0.653	<0.001
Lipid-lowering drugs^19^,^24^	136	128	1.06 (0.84–1.35)	0.50	0.617	0.169	47.1
Alcohol^20^,^22^,^23^,^25^,^26^	45	52	0.86 (0.58–1.28)	0.73	0.466	0.908	<0.001
Diabetes^19^,^22^,^24^,^25^	175	171	1.02 (0.83–1.26)	0.22	0.829	0.477	<0.001
Current smoker^19^,^20^,^22^,^26^	181	180	1.01 (0.82–1.23)	0.05	0.962	0.727	<0.001
Hypertension^19^,^22^,^24^	154	150	1.03 (0.82–1.28)	0.23	0.814	0.289	19.4

CVD, cardiovascular disease; CRC, colorectal cancer.

*P*, *P* value for association.

*P*_*h*_, *P* value for heterogeneity.

^a^only articles reporting the variables were analyzed.

^b^weighted mean of included articles.
