# Supplementary material for: Folic acid supplements and colorectal cancer risk: meta-analysis of randomized controlled trials
Source: Sci Rep. 2015 Jul 1;5:12044. doi: 10.1038/srep12044 (PMC4487230; doi:10.1038/srep12044)
Supplement: Supplementary Table S1 [file srep12044-s1.pdf]

Folic acid supplements and colorectal cancer risk: meta-analysis of randomized controlled trials

Tingting Qin[1☯], Mulong Du[2,3☯], Haina Du[1], Yongqian Shu[1], Meilin Wang[2,3*], Lingjun Zhu[1*]

[1]Department of Oncology, The First Affiliated Hospital of Nanjing Medical University, Nanjing, China.

[2]Department of Environmental Genomics, Jiangsu Key Laboratory of Cancer Biomarkers, Prevention and Treatment, Cancer Center, Nanjing Medical University, Nanjing, China.

[3]Department of Genetic Toxicology, the Key Laboratory of Modern Toxicology of Ministry of Education, School of Public Health, Nanjing Medical University, Nanjing, China.

☯ These two authors contributed equally to this work.

[*] Corresponding author:

Lingjun Zhu: Department of Oncology, The First Affiliated Hospital of Nanjing Medical University, 300 GuangZhou Rd, Nanjing 210029, Nanjing, China; Cell phone: (0)13951807457; E-mail address: zhulingjun@njmu.edu.cn; or

Meilin Wang: Department of Environmental Genomics, School of Public Health, Nanjing Medical University, 818 East Tianyuan Road, Jiangning District, Nanjing 211166, China. Tel: +86-25-86868423; Fax: +86-25-86862684. E-mail address:

mwang@njmu.edu.cn

Supplementary Table S1. Assessments for the eligible studies.

Features used to assess quality of trial reports	Enrolled studies (Author / Publication year)							
	Lonn *et al.* / 2006	Cole *et al.* / 2007	Logan *et al.* / 2008	Zhang *et al.* / 2008	Wu *et al.* / 2009	Armitage *et al.* / 2010	Hankey *et al.* / 2012	Gao *et al.* / 2013
1. Was the study described as randomized?	Y	Y	Y	Y	Y	Y	Y	Y
2. Was the study described as double-blind?	Y	Y	Y	Y	Y	Y	Y	N
3. Did the trial report the following methods for generating the allocation sequence: computer, random numbers table, shuffled cards or tossed coins?	Y	Y	Y	U	Y	Y	Y	Y
4. Did the trial report the numbers and reasons for withdrawal?	Y	Y	Y	Y	Y	Y	Y	Y
5. Did the trial report allocation concealment?	Y	Y	Y	U	Y	Y	Y	Y

Y for "Yes", N for "No", U for "Unclear"
